# Young men’s gambling and violence perpetration in Mwanza, Tanzania

**DOI:** 10.1093/eurpub/ckac129.057

**Published:** 2022-10-25

**Authors:** H Stöckl, R Brambilla, G Mshana, D Malibwa, S Sichalwe, S Kapiga

**Affiliations:** 1 Ludwig-Maximilians-Universität München, München, Germany; 2 National Institute for Medical Research, Mwanza, Tanzania; 3 Mwanza Intervention Trials Unit, Mwanza, Tanzania; 4 LSHTM, London, UK

## Abstract

**Background:**

The prevalence of intimate partner violence (IPV) in Tanzania is one of the highest in the sub-Saharan African region. Studies have shown that traditionally “manly” behaviours, such as risk-taking, are at the root of IPV perpetration. Only few studies investigated the co-occurrence of gambling and IPV, and none from LMICs.

**Methods:**

Cross-sectional survey data of 1002 men aged 18-24 from Mwanza, Tanzania were analysed. Physical, sexual, emotional and economic IPV perpetration were measured using acts-based questions. Gambling was assessed through a question on whether the man bet or spent money on gambling or gambling machines. Consequences of gambling behaviours were assessed through four further questions. We conducted multivariate logistic regressions to control for potential confounders.

**Results:**

21% of the men in the sample confirmed they had bet or spent money on gambling in the previous 12 months; the prevalence raises to 24% for men who had been in a relationship in the previous 12 months (N = 755). Of these, 23% had ever perpetrated physical IPV, 29% sexual IPV, 56% emotional IPV and 37% economic IPV in their lifetimes. Of those who gambled, 24% had ever perpetrated physical IPV, 46% ever committed sexual IPV, 66% emotional IPV and 45% economic IPV. Gambling was statistically significantly associated sexual IPV (aOR: 2.39; 95% CI: 1.66-3.45) and emotional IPV (aOR: 1.48; 95% CI: 1.03-2.14) even after controlling for age, alcohol use, depressive symptoms and suicidal ideation. Gambling was not associated with physical and economic IPV after adjusting for those confounders.

**Implications:**

The analysis shows that young men's practice of gambling is an additional risk factor for IPV perpetration that needs to be addressed. More research is needed to understand how current prevention efforts can be expanded to include problem gambling treatment to curb the incidence of IPV and give couples conflict resolutions skills for issues that might arise from gambling.

**Key messages:**

• Problem gambling has so far remained vastly under-researched in violence research.

• Gambling as well as drinking were associated with increased odds of physical and sexual IPV perpetration.

